# Safety and tolerability of a low glycemic load dietary intervention in adults with cystic fibrosis: a pilot study

**DOI:** 10.3389/fnut.2024.1441201

**Published:** 2024-09-25

**Authors:** Kevin J. Scully, Laura Truex, Alina Brennan, Robert Fowler, Gregory S. Sawicki, Ahmet Uluer, David S. Ludwig, Cara B. Ebbeling, Leah Langlais, Angela Dziok, Steven D. Freedman, Melissa S. Putman

**Affiliations:** ^1^Division of Endocrinology, Hasbro Children’s Hospital, Providence, RI, United States; ^2^Division of Gastroenterology, Hepatology, and Nutrition, Boston Children’s Hospital, Boston, MA, United States; ^3^Division of Pulmonary Medicine, Boston Children’s Hospital, Boston, MA, United States; ^4^Division of Pulmonary and Critical Care Medicine, Brigham and Women’s Hospital, Boston, MA, United States; ^5^New Balance Foundation Obesity Prevention Center, Boston Children's Hospital, Boston, MA, United States; ^6^Cystic Fibrosis Center, Rhode Island Hospital, Providence, RI, United States; ^7^Division of Gastroenterology, Beth Israel Deaconess Hospital, Boston, MA, United States; ^8^Diabetes Research Center, Massachusetts General Hospital, Boston, MA, United States

**Keywords:** cystic fibrosis, nutrition, diet, body composition, continuous glucose monitoring

## Abstract

**Introduction:**

To achieve and maintain adequate weight, people with cystic fibrosis (CF) May often consume energy-dense, nutrient-poor foods high in added sugars and refined carbohydrates; however, little is known about the glycemic and metabolic effects of dietary composition in this patient population. The objective of this pilot study was to investigate the safety and tolerability of a low glycemic load (LGL) diet in adults with CF and abnormal glucose tolerance (AGT).

**Methods:**

Ten adults with CF and AGT completed this prospective, open-label pilot study. Mean age was 27.0 ± 2.1 years, 64% were female, and all had pancreatic insufficiency. Each participant followed his/her typical diet for 2 weeks, then transitioned to a LGL diet via meal delivery service for 8 weeks. The primary outcome was change in weight from baseline to study completion, with safety established if no significant decline was noted. Other key safety outcomes included change in hypoglycemia measured by patient report and continuous glucose monitoring (CGM). Exploratory outcomes included changes in other CGM measures, body composition by dual energy X-ray absorptiometry (DXA), and patient reported outcomes.

**Results:**

There were no significant changes in weight or in subjectively-reported or objectively-measured hypoglycemia. Favorable non-significant changes were noted in CGM measures of hyperglycemia and glycemic variability, DXA measures of fat mass, and gastrointestinal symptom surveys.

**Discussion:**

A LGL dietary intervention was safe and well tolerated in adults with CF and AGT. These results lay the groundwork for future trials investigating the impact of low-glycemic dietary interventions on metabolic outcomes in the CF population.

## Introduction

Maintenance of a healthy body mass index (BMI) is associated with reduced morbidity and mortality in persons with cystic fibrosis (PwCF) (1, 2). Because of this, CF nutritional guidelines recommend maintaining a BMI at or above the 50%ile for age for children and adolescents, ≥22 kg/m^2^ in adult females and ≥ 23 kg/m^2^ in adult males. To achieve and maintain adequate weight, pwCF are encouraged to consume a caloric intake of 120–150% of the dietary reference intake (DRI) for the typical healthy adult (3–7). Historically, this concern for malnutrition often led providers to recommend high-calorie diets without concern for dietary quality, resulting in a tendency for PwCF to overconsume energy-dense, nutrient-poor foods, particularly foods high in added sugars and refined carbohydrates (8–10). High-carbohydrate intake May lead to complications such as dysglycemia (particularly early post-prandial hyperglycemia and late post-prandial hypoglycemia), inflammation, and gastrointestinal dysmotility, predisposing to obesity, metabolic syndrome and cardiovascular disease (11).

Dietary recommendations for children and adults with CF are based primarily on consensus and expert opinion. Current guidelines do not specify the composition of carbohydrate intake apart from avoiding artificial sweeteners and closely monitoring carbohydrate intake to maintain glycemic control (2, 5–7). Dietary changes are commonly used for treatment of CF-related diabetes (CFRD), which affects up to half of adults with CF, despite the lack of efficacy data (12). Thus, research is needed to determine whether modifiable dietary factors could aid in the management of dysglycemia and prevention of associated complications (10, 13, 14).

Glycemic index (GI) quantifies the rise in blood glucose during the first two hours after consumption of a food or meal, reflecting the rate by which carbohydrates are digested and/or metabolically transformed into glucose. Glycemic load (GL), the multiplicative product of GI and the amount of carbohydrate consumed, reflects how blood glucose is affected by standard portions of carbohydrate-containing foods. Diets with a low GI (LGI) and low GL (LGL) May include non-starchy vegetables, legumes, minimally processed grains, and temperate fruits, with reduction of refined grains, starchy vegetables, fruit juices, and concentrated sweeteners. A LGL diet also typically incorporates a reduction in total dietary carbohydrate. In people with both type 1 and type 2 diabetes mellitus, a LGL diet has been shown to improve glycemic variability, HbA1c levels, insulin sensitivity, and quality of life without increasing hypoglycemic events (15–18).

Few studies have prospectively evaluated the impact of dietary quality on glycemic control and body composition in PwCF. Feeding trials, where participants live in confined quarters with close monitoring to ensure adherence, have higher internal validity but lower external validity compared to trials relying on nutrition education and dietary counselling. When assessing dietary interventions under free-living conditions, food delivery can enhance adherence and increase the rigor of trials aimed at evaluating safety and efficacy. As PwCF live longer with highly effective modulator therapy (HEMT) and as the prevalence of obesity, cardiovascular disease, and metabolic syndrome increases in PwCF (19–24), it is crucial to understand the effects of dietary composition on short- and long-term endocrine, gastrointestinal, and pulmonary outcomes.

The goal of this pilot study was to determine the safety and tolerability of an LGL diet provided via home delivery on dysglycemia and body composition in adults with CF and abnormal glucose tolerance (AGT). We hypothesized that an LGL diet over an eight-week period would be well tolerated and associated with weight maintenance in adults with CF.

## Materials and methods

### Study design and setting

This was a 10-week prospective, open-label study beginning with 2 weeks of typical diet followed by 8 weeks of the LGL dietary intervention provided via home meal delivery service. Study visits took place in the Boston Children’s Hospital/Brigham and Women’s Hospital (BCH/BWH) CF Center. The study was approved by the Boston Children’s Hospital Institutional Review Board (IRB) and registered on clinicaltrials.gov (NCT#04529853). Written informed consent was obtained from all participants.

### Study population

Study enrollment took place between October 2021 and May 2023. Participants were recruited from the BCH/BWH CF Center. Eligibility criteria included adults aged 18–70 years with an established diagnosis of CF, pancreatic insufficiency, and a history of documented abnormal glucose tolerance, either impaired glucose tolerance (IGT) or indeterminate glycemia (INDET; 1-h glucose >200 mg/dL). Exclusion criteria included pregnancy, history of solid organ transplantation, FEV1 < 50%, BMI <20 or > 28 kg/m^2^, enteral nutrition dependence, use of antibiotics or systemic supraphysiologic glucocorticoids within 1 month, initiation of a cystic fibrosis transmembrane conductance regulator (CFTR) modulator within 3 months, current adherence to a LGL or other carbohydrate-restricted diet (carbohydrates <30% total daily caloric intake), current use of insulin, or a diagnosis of CFRD (HbA1c ≥6.5% or 2-h OGTT glucose ≥200 mg/dL). The diagnoses of IGT, INDET and CFRD were confirmed by chart review, including OGTT and HbA1c levels obtained within the preceding 2 years, using the criteria established by both the American Diabetes Association (ADA) and Cystic Fibrosis Foundation (CFF) (3). These BMI criteria were chosen to avoid who might be at risk for becoming underweight if weight loss occurred during the study period, and also to include those with mild overweight as this would be unlikely to impact glycemia while also reflect the increasing frequency of mildly elevated BMI values observed in the post-ETI era.

### Clinical assessments

Study visits took place at baseline and after completion of the 8-week feeding period (10 weeks). At each visit, anthropometric data were collected, and participants completed questionnaires regarding quality of life (QoL, Cystic Fibrosis Questionnaire-Revised [CFQ-R]), gastrointestinal symptoms (Patient Assessment of Constipation [PAC-SYM] Score (25), Patient Assessment of Gastrointestinal Symptoms [PAGI-SYM] Score (26), Bristol Stool Chart), and activity level (Modified Activity Questionnaire [MAQ] score) (27). After study completion, participants also completed a non-validated questionnaire regarding LGL diet tolerability, which included 5 questions assessing diet satisfaction, hunger, diet ease, taste, and diet recommendation likelihood using a 10-point Likert scale (Supplementary Figure S1).

Baseline clinical characteristics were obtained by questionnaire and included medical history, pancreatic insufficiency (defined as pancreatic enzyme replacement requirement), medications, hospitalizations, and pulmonary exacerbations over the past year. Race and ethnicity were self-reported. Height and weight were measured on a calibrated wall-mounted stadiometer and electronic scale, respectively. Medical records were reviewed for CF*TR* genotype, confirmation of pulmonary exacerbations, and recent spirometry results (within 3 months), including percent predicted forced expiratory volume in 1 s (FEV1) and forced vital capacity (FVC).

### Dietary intervention

After enrollment, participants continued their typical diet with no restrictions on carbohydrate consumption or sugar content for 14 days. Dietary intake data were collected by the study dietitian twice during the first week of the typical diet period and analyzed using the Nutrition Data System for Research software version 2022, developed by the Nutrition Coordinating Center (NCC), University of Minnesota, Minneapolis, MN. The study dietitian then determined minimum caloric requirement for each participant utilizing the Harris-Benedict (HB) equation to estimate basal metabolic rate (BMR). The BMR was multiplied by a stress factor (SF) between 1.4 and 2.0 to account for the caloric demands of activities of daily living (ADL), physical activity, and disease severity. The study dietician then cross-checked these calculated values with each participant’s dietary intake data obtained during the typical diet period and used the higher of these values as the minimum caloric requirement for the LGL dietary intervention period.

After the typical diet period, each participant transitioned to a LGL diet (macronutrient breakdown: carbohydrate 30%, fat 50%, protein 20%; >90% food items with glycemic load <55) for the next 8 weeks (Supplementary Figure S2). Pancreatic enzyme replacement therapy (PERT) was adjusted by study physicians to account for changes in dietary fat. All meals and snacks were provided by the food delivery service Metabolic Meals™. Participants were encouraged to consume only those foods delivered via the delivery service, however also received educational materials and a detailed list of acceptable food items to account for real-world dietary challenges. Consumption of water and sugar-free beverages was unrestricted. Study staff communicated with each participant on a weekly basis to encourage dietary adherence, query instances of hypoglycemia symptoms, and address any questions.

### Laboratory and glycemic measures

At each study visit, participants underwent blood draw for HbA1c, erythrocyte sedimentation rate (ESR), and C-reactive protein (CRP) levels. HbA1c levels were measured using an NGSP-certified instrument.

Each participant received education about the signs and symptoms of hypoglycemia and was provided a glucometer to check fingerstick blood glucose values if these symptoms occurred. A blinded CGM sensor (Dexcom G6 Pro, Dexcom Inc., San Diego CA, mean absolute relative difference 9% (28)) was placed at the initial study visit, and participants were taught how to change sensors every 10 days. Sensors were mailed back to the study team, and data were uploaded to the Dexcom Clarity application.

### Weight and body composition

Participants underwent whole body dual-energy X-ray absorptiometry (DXA) scans for body composition analyses at baseline and study completion (Hologic Horizon densitometer, Hologic Inc., Bedford, MA). Participants were provided a calibrated digital scale at baseline and reported home-measured weight on a weekly basis.

### Statistical analysis

Statistical analyses were performed using STATA (version 16, 2019; College Station, TX: StataCorp LLC). All tests were two-sided, and *p* < 0.05 was considered statistically significant. The primary outcome measure was change in weight from baseline to study completion, with safety established if no significant decline in weight was noted from baseline to study completion. Secondary outcomes included change in key CGM measures (average glucose [AG], standard deviation [SD], coefficient of variation [CV], % time in range 70–180 mg/dL [TIR], % tighter time in range [70–140], % time > 140 mg/dL, >180 mg/dL, >250 mg/dL, <70 mg/dL and < 54 mg/dL), DXA-derived body composition measures, biochemical values (HbA1c, ESR, CRP), and questionnaire data. The normality of the paired difference for each primary and secondary outcome was assessed with the Shapiro–Wilk test. Paired t-tests and the Wilcoxon signed rank test were used for outcomes with normally and non-normally distributed data, respectively.

The key safety concern with this dietary intervention was weight loss, which can be detrimental in CF; therefore, the study was powered to detect a clinically significant decline in weight over the 10-week study period. Sample size calculations were based on prior published data estimating average expected weight variability over a 3-month period without dietary or other medical intervention in adults with CF (1.68 ± 1.71 kg) (29). Based on these data, a sample size of 10 patients would be needed to have an 80% power to detect a clinically significant weight difference using a two-sided test with 5% type 1 error. Given that this was a pilot and feasibility study, secondary outcomes were exploratory and not considered in power calculations.

## Results

### Participant flow and baseline characteristics

Eleven participants were enrolled, 10 of whom completed the study (Supplementary Figure S1). Upon enrollment, 5 participants had evidence of pre-diabetes (HbA1c 5.7–6.4%), 8 participants had IGT on OGTT, and 2 participants met criteria both for pre-diabetes and IGT. No participant was enrolled purely with INDET. The average time between eligibility data and enrollment was 10.9 months (median 9 months, range 0–22.8 months). Four of the 11 participants had enrollment data older than 12 months. Baseline clinical characteristics of all participants (*n* = 11) are summarized in Table 1. Mean age was 27 ± 2 years (range 19–45 years). Seven participants (63.6%) were female, 9 (82%) had at least one copy of the F508del mutation, and all had a history of exocrine pancreatic insufficiency. All but one participant was taking a CFTR modulator (90.9%), the majority of whom were taking highly effective CFTR modulator therapy (elexacaftor-tezacaftor-ivacaftor, ETI). None of the participants initiated or changed modulator therapy in the 1-year preceding enrollment. The average percent predicted forced expiratory volume in 1 s (FEV1) was 86%, BMI 24 kg/m^2^, and HbA1c 5.6%. One participant dropped out of the study <1 week into the dietary intervention phase due to study-independent personal concerns, and the remainder completed the dietary intervention. Of the remaining 10 participants, 8 provided DXA body composition data at baseline and study completion.

**Table 1:** Summary statistics.

	*n* = 11
**Age (years)**	27.0 ± 2.1
**Female, *n* (%)**	7 (63.6%)
**Race, *n* (%)**	
Caucasian	11 (100%)
**Ethnicity, *n* (%)**	
Hispanic Non-Hispanic	1 (9.1%) 10 (90.9%)
**Height (cm)** Females Males	163.6 ± 3.0 157.9 ± 2.0 173.5 ± 2.0
**Weight (kg)**	64.5 ± 3.0
**BMI (kg/m** ^**2**^ **)**	24.0 ± 0.62
**Genotype, *n* (%)**	
F508del homozygous F508del heterozygous Other	6 (54.5%) 3 (27.3%) 2 (18.2%)
**Pancreatic insufficiency, *n* (%)**	11 (100%)
**FEV1 (% predicted)**	86 ± 6
**FVC (% predicted)**	96 ± 5
**HbA1c**	5.6 ± 0.1
**CF liver disease**	4 (36.4%)
**Modulator use, *n* (%)** Elexacaftor/tezacaftor/ivacaftor Ivacaftor	10 (90.9%) 9 (81.8%) 1 (9.1%)
**Baseline nutrition data (% daily value)**	
Total calories (kcal) Carbohydrate (g) Fat (g) Protein (g) % calories from carbohydrate % calories from fat % calories from protein Total sugars (g) Added sugars (g)	2,975 329 (120%) 130 (167%) 131 44% 38% 18% 139.5 86 (171%)

Data displayed as mean ± standard error of the mean or n (%) unless otherwise stated.

FEV1, forced expiratory volume in 1 s; FVC, forced vital capacity; HbA1c, hemoglobin A1c.

Baseline nutrition data from participants’ run-in period showed a macronutrient composition of 44% of energy from carbohydrate, 38% from fat and 18% from protein. Carbohydrate intake (120%) and added sugar (171%) exceeded recommendations for the general population (Table 1).

### Weight and body composition

From baseline to study completion, there were no significant changes in weight (mean 64.8 vs. 64, median 62 vs. 61.5, *p*-value 0.26) or BMI (mean 24.1 vs. 23.8, median 24.3 vs. 23.6, p-value0.23) (Table 2). No participant experienced a weight change >7%.

**Table 2:** Changes in glycemic and body composition measures from baseline to post-LGL diet.

	Baseline	Post-LGL diet	*p*-value
**Body composition**			
Weight (kg)	64.8 ± 3.3 (57.2, 72.3)	64.0 ± 3.2 (56.9, 71.2)	0.26
BMI (kg/m^2^)	24.1 ± 0.6 (22.6, 25.5)	23.8 ± 0.6 (22.5, 25.1)	0.23
Total fat mass (gm)	23,159 ± 2,681 (16,818, 29,498)	22,133 ± 2,848 (15,398, 28,869)	0.11
Total lean mass (gm)	43,545 ± 3,868 (34,398, 52,691)	43,552 ± 3,980 (34,138, 52,965)	0.99
% fat mass	34 ± 3.7 (25.4, 42.7)	33 ± 3.9 (23.7, 42.4)	0.09
Trunk fat (gm)	10,294 ± 1,449 (6,867, 13,720)	9,805 ± 1,502 (6,253, 13,357)	0.14
% trunk fat	30.7 ± 3.7 (22, 39.4)	29.8 ± 4.0 (20.3, 39.3)	0.18
Fat mass index (FMI, kg/m^2^)	8.6 ± 1.1 (6.1, 11.1)	8.2 ± 1.1 (5.6, 10.9)	0.16
Lean mass index (LMI, kg/m^2^)	15.8 ± 0.8 (14, 17.5)	15.8 ± 0.8 (13.8, 17.8)	0.68
Appendicular lean mass index (ALMI, kg/m^2^)	6.5 ± 0.5 (5.4, 7.6)	6.6 ± 0.5 (5.5, 7.6)	0.55
**Glycemic measures**			
HbA1c (%)	5.7 ± 0.2 (5.2, 6.3)	5.6 ± 0.2 (5.2, 6.1)	0.55
AG (mg/dL)	127.1 ± 5.5 (114.8, 139.5)	120.7 ± 3.1 (113.7, 127.7)	0.16
SD (mg/dL)	29.8 ± 2.3 (24.6, 35.1)	28.5 ± 1.5 (25.1, 31.9)	0.96
CV (%)	23.2 ± 1.0 (21, 25.4)	23.6 ± 1.1 (21.1, 26.2)	0.58
% time < 54 mg/dL	0.2 ± 0.1 (0.05, 0.4)	0.5 ± 0.2 (0.05, 0.9)	0.36
% time < 70 mg/dL	1.0 ± 0.5 (0.1, 2)	1.4 ± 0.4 (0.4, 2.4)	0.28
% time 70–180 mg/dL	91.4 ± 2.6 (85.6, 97.2)	94.2 ± 0.9 (92.2, 96.2)	0.48
% time 70–140 mg/dL	74.2 ± 5.3 (62.3, 86)	78.8 ± 5.4 (73.2, 84.4)	0.51
% time > 180 mg/dL	7.6 ± 2.7 (1.6, 13.7)	4.4 ± 0.8 (2.5, 6.4)	0.17
% time > 140 mg/dL	24.8 ± 2.5 (12.6, 37.1)	19.8 ± 2.5 (14.1, 25.5)	0.28
% time > 250 mg/dL	1.1 ± 0.5 (0.2, 2.3)	0.3 ± 0.1 (0.03, 0.6)	0.39
**Questionnaire data**			
**PAGI**	13.3 ± 2.6 (7, 19.6)	8.7 ± 6.3 (0, 24.2)	0.07
**PAC**	13.3 ± 5.7 (0.7, 27.2)	5.3 ± 1.9 (0.7, 9.9)	0.16
**Bristol**	3.7 ± 0.6 (2.1, 5.3)	3.6 ± 0.4 (2.7, 4.5)	0.87
**MAQ**	11,386 ± 6,188 (0, 25,818)	12,358 ± 9,735 (0, 39,431)	0.72

Data displayed as average ± standard error (95% confidence interval).

Weight, BMI, CGM measures, *n* = 10; DXA measures, *n* = 8; Questionnaire data *n* = 7.

LGL, low glycemic load; BMI, body mass index; AG, average glucose; SD, standard deviation; CV, coefficient of variation; PAGI, patient assessment of gastrointestinal symptoms; PAC, patient assessment of constipation; MAQ, modified activity questionnaire.

**Figure 1:**
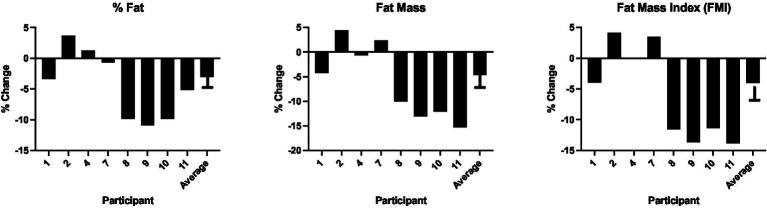
Percent change in % fat, fat mass and FMI pre- and post-LGL diet percent change in key DXA body composition metrics for each participant from study baseline to completion, as well as the average change across the entire study cohort.

DXA body composition analyses showed non-significantly reduced fat mass, percent fat, and fat mass index after the LGL dietary intervention (*n* = 8; Table 2). With exclusion of one participant who significantly decreased activity level during the dietary intervention phase, the remaining cohort (*n* = 7) showed significant reductions in % fat mass, total fat mass, trunk fat mass, and % truncal fat (Supplementary Table S1). Figure 1 depicts the individual and average changes in % fat and fat mass pre- and post-LGL diet.

### Glycemic outcomes

**Figure 2:**
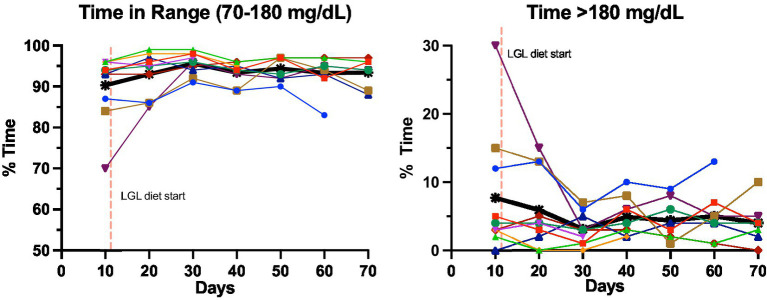
Change in glycemic measures pre- and post-LGL diet change in key CGM measures from baseline (weeks 1–2) to post-LGL diet completion (week 10). Each study participant is represented by a different color. Thicker black lines represent the average across all participants.

During the dietary intervention phase, there were no subjective episodes of hypoglycemia reported by participants, nor were any significantly low fingerstick glucose values identified (<54 mg/dL). HbA1c did not change over the course of the study period (mean 5.7% vs. 5.65%, median 5.5% vs. 5.7%, *p* = 0.55, Table 2). Similarly, there were no changes in CGM-measured hypoglycemia pre- and post-LGL dietary intervention (% time < 54: mean 0.3% vs. 0.5%, median 0.2% vs. 0.2%, *p* = 0.36; % time < 70: mean 1% vs. 1.4%, median 0.6% vs. 1.2%, *p* = 0.28, Table 2). Several CGM measures showed non-significant changes favoring the LGL dietary intervention phase, including reductions in AG, SD, % time > 180, % time > 140, % time > 250 mg/dL and increase in % TIR and %TTIR (Table 2). Figure 2 displays the individual and average changes in TIR and % time > 180 mg/dL throughout the study duration.

### Other exploratory outcomes

No significant changes were noted in inflammatory markers (ESR, CRP) or questionnaire data, though there were favorable non-significant improvements in self-reported gastrointestinal symptoms (PAC, PAGI SYM; Table 2). Of the 8 participants with complete questionnaire data, 7 (87.5%) had improvement in PAGI-SYM score and 8 (100%) in PAC score. There were no significant changes in CFQ-R scores (data not shown). In terms of diet tolerability, 6 of 8 (75%) reported overall satisfaction with the diet, 5 (63%) felt that the diet was easy to follow, and 7 (88%) would overall recommend the diet to others (Supplementary Table S1). After completion of the LGL diet, participants reported an average tolerability score of 33 out of 50 total points (Table 2). None of the participants stopped the dietary intervention early due to side effects or intolerance.

## Discussion

In this prospective, open-label pilot study, a LGL dietary intervention was safe and well tolerated over 8 weeks in PwCF and AGT, with no evidence of significant weight loss or subjectively-reported or objectively-measured hypoglycemia. Non-significant changes toward improvements in CGM-derived measures of hyperglycemia, DXA measures of fat mass, and gastrointestinal symptom scores were noted after the LGL diet, though the small sample size likely limited the power to detect significant changes in these exploratory outcomes. To our knowledge, this is the first study utilizing a standardized meal delivery service to prospectively evaluate the impact of dietary modification on weight, body composition, and dysglycemia in PwCF. These results support future larger, longer interventional studies investigating dietary quality in this patient population.

Nutritional optimization has long been a focus of care in PwCF, as maintaining a healthy BMI has been associated with improved lung function, clinical outcomes, and overall survival (1–3, 30). The Academy of Nutrition and Dietetics, CFF, ADA, and European Society for Clinical Nutrition and Metabolism (ESPEN) recommend utilizing the same dietary macronutrient percentages as recommended for the general population (40–50% calories from carbohydrate, 35–50% from fat, and 20% from protein) (3, 5, 31). However, these recommendations are built on general consensus rather than evidence and do not specify the type of carbohydrate (2, 5–7). Historically, many PwCF have tended to maintain BMI by consuming energy dense but nutritionally devoid food items (14, 32). For example, a cross-sectional study comparing 80 children ages 2–18 years with CF and gender- and age-matched controls found that children with CF consumed significantly more energy-dense, nutrient poor foods, as well as a higher number of sugar-added beverages (8). We previously conducted a cross-sectional analysis in 38 adolescents and adults with CF compared to 19 gender- and age-matched controls and found that participants with CF consumed significantly higher GI foods, a greater proportion of calories from fat, and a lower proportion from protein (10). Baseline nutrition data from our current study showed notably higher intake of carbohydrates and added sugars than the recommended percent daily value.

The introduction of HEMT has changed the nutritional landscape of CF. HEMT has been associated with significant weight gain occurring within the first 6 months of initiation and has contributed to rising rates of overweight and obesity in people with CF after FDA approval in the fall of 2019 (24, 32, 33). According to the 2022 CF Foundation Patient Registry Annual Report, 40.9% of adults with CF met criteria for either overweight or obesity (44.6% men, 36.9% women) (12). Nutrition studies investigating dietary quality in the post-modulator era are critical to guide the development of a new approach for optimizing not only BMI but also other important outcomes including body composition and glycemia.

Studies investigating the relationship between macronutrient distribution and dysglycemia in PwCF are limited and show varied results. One study in 36 adults with CF and a broad range of glycemia (NGT, AGT, and CFRD) compared 3-day self-reported dietary recall data to CGM data collected over the same period, finding no association with macronutrient content or total energy and measures of glycemic variability including SD, CV, mean amplitude of glycemic excursions (MAGE); however, carbohydrate quality, GI, and GL were not assessed (13). On the other hand, another study in 18 adults with CF without CFRD found that SD and MAGE were positively correlated with carbohydrate content, sugar, added sugar, and GL. Percent time > 7.8 mmol/L (140 mg/dL) was correlated with GI, while % time 3.9–7.8 mmol/L (70–140) was negatively correlated with added sugar, GI and GL (14). One prospective study compared fasting glucose, HbA1c, and triglyceride levels in 44 children and adolescents with CF randomized to receive nutritional instruction for either a high-fat, high-calorie (HFHC) diet or a low-glycemic HFHC diet over a 3-month period (34). Children receiving instruction on a low-glycemic diet had a significant decrease in fasting glucose, HbA1c and triglyceride levels as well as a significantly greater increase in weight; however, this study was conducted under free-living conditions without any measures of dietary adherence. Additionally, GI was measured via participant-reported nutrition logs, CGM was not utilized, and significant drop-out occurred in this study (34), limiting generalizability of the results. There are no prior published dietary intervention studies investigating glycemic outcomes that have utilized home delivery of meals in CF.

In our study, we identified favorable non-significant changes in CGM measures of hyperglycemia, even though none of our participants had CFRD and our sample size was relatively small. Most participants had HbA1c levels that were either normal or in the range of pre-diabetes; given that the dietary intervention phase of our study took place over only 8 weeks, the lack of meaningful changes in HbA1c in our population is not surprising.

While insulin is currently the only known effective treatment option for CFRD, it has not yet proven to be clinically beneficial for individuals with CF who have AGT (3, 14). AGT has been associated with earlier progression to CFRD and poorer clinical outcomes (35–37). Although the size of our cohort and relatively short study period cannot address the long-term effect of a LGL diet on key clinical outcomes and/or CFRD progression, these results highlight the need for long-term prospective studies designed to investigate the impact of dietary modification on future development of CFRD and pulmonary decline.

Data from our current study showed no significant changes in weight, BMI or lean mass over the 8-week intervention period, but did show several favorable non-statistically significant decreases in multiple measures of fat mass. While BMI has classically been the primary measure of nutritional outcomes in PwCF, there is interest in evaluating other potentially more meaningful predictors of health status in CF that better distinguish between fat and lean mass. Many centers are starting to utilize DXA and/or bioelectrical impedance analysis (BIA) to measure body composition in PwCF (10, 38–40). Our body composition results are particularly noteworthy given that prior data in PwCF have shown positive correlations between lung function and measures of lean mass and negative correlations with measures of fat mass (% fat, % truncal fat, fat mass index [FMI, kg/m2]) when adjusting for age, gender and BMI (9, 10, 38, 40–42). While we cannot draw definitive conclusions from these preliminary results, it is certainly possible that a diet that only partially restricts carbohydrate consumption and instead focuses more on carbohydrate quality would minimize the risk of significant weight loss by preserving lean mass, potentially leading to clinically advantageous body composition changes over the long-term for PwCF. Larger, long-term studies are needed to answer this question.

Up to 85% of people with CF experience gastrointestinal symptoms, including malabsorption, gastroesophageal reflux (35–81%), gastroparesis (38%), chronic abdominal pain (60% children, 36% adults), constipation (47%), distal intestinal obstruction syndrome (~16%), and small intestine bacterial overgrowth (30–50%) (43). Some have theorized that high-GI carbohydrates cannot be metabolically used by gut microbes, resulting in decreased production of gut-microbiota-generated short chain fatty acids, and shift from an anti- to pro-inflammatory state (44). The majority of our participants had normal baseline inflammatory markers; therefore, we did not detect any notable changes in these values. However, our study did find non-significant changes toward improvement in gastrointestinal symptoms via validated questionnaires, though these values were not statistically significant. The fact that symptom scores did not worsen over the course of the study despite the substantial change in dietary composition is also reassuring.

Strengths of this study include the comprehensive, prospectively measured clinical, DXA, and CGM measures, as well as utilization of a meal home delivery service for optimal dietary adherence. However, several important limitations of this study should be noted. Participants were recruited from a single study center and geographical region with limited racial and ethnic representation. Participants enrolled in this study were relatively healthy with mild impairments in lung function; individuals with more advanced lung disease May have a different clinical response to a dietary intervention. While this pilot study was appropriately powered for our primary outcome, the sample size was small, thus limiting detection of any statistically significant changes in exploratory outcome measures such as CGM measures and body composition. Three individuals did not complete a follow-up DXA analysis, which likely further limited the power to detect changes in body composition results. The time period between DXA scans was also relatively short (2 months); however, studies in other patient populations have found that DXA analysis can accurately detect changes in body composition over time intervals such as this (45, 46). In addition, physical activity was measured via participant-reported surveys and not by a wearable activity tracker or fitness monitor. While meal delivery significantly improves dietary intervention compliance, we cannot definitively know if all participants strictly adhered to the dietary intervention throughout the entire study duration. However, participants received nutrition education and materials about acceptable LGL food items to account for this possibility. Utilization of a live-in/residential model would be the most ideal study design to ensure strict dietary adherence but was cost-prohibitive and logistically unfeasible for this study. Our study focused on changes in glycemia, not on beta-cell function or insulin secretory capacity. Mixed meal tolerance testing can be used in future larger studies to assess the impact of LGL diet on pancreatic endocrine function. Given the timing of the COVID-19 pandemic, many potential participants were lacking typical annual glycemic screening data, leading us to use a longer inclusion timeframe for our enrollment criteria (HbA1c or OGTT data within 2 years). This lack of data is in line with the 2022 CFF patient registry report, which showed only around 30% of adults with CF underwent recommend annual OGTT for diabetes screening. Given the known inter-variability in glucose tolerance among PwCF, it is possible that glycemic status in those individuals with older data May have changed. Similarly, recent data regarding cholesterol levels and hepatic function were missing on the majority of participants at baseline, therefore we could not assess the potential effect of a higher-fat diet on these metrics. Given the small sample size of this study, we did not have the power to stratify participants based on characteristics such and duration of CF-related dysglycemia or pancreatic enzyme replacement. Lastly while we did not include a control group in our pilot study, these data do support the need for a larger randomized control trial investigating nutritional intervention in PwCF.

In conclusion, this pilot study found that an 8-week LGL dietary intervention was safe and well tolerated in PwCF and AGT, with no evidence of significant weight loss or increase in hypoglycemia. Despite the small sample size, there were several favorable non-significant changes toward improvements in body composition, glycemia, and gastrointestinal symptoms. While this was intended primarily as a feasibility study, limiting conclusions that can be drawn, these results provide critical preliminary data to inform prospective long-term interventional studies designed to more comprehensively test the impact of a LGL diet on key clinical outcomes in PwCF, including those with more severe dysglycemia and CFRD. Particularly as rates of metabolic syndrome and CFRD in PwCF continue to increase, there is a great need for studies investigating the role of dietary quality to provide rigorous, evidence-based data guiding nutrition recommendations in the post-modulator era.

## Data Availability

The datasets presented in this study can be found in online repositories. The names of the repository/repositories and accession number(s) can be found in the article/Supplementary material.
