# Supplementary material for: Safety and tolerability of a low glycemic load dietary intervention in adults with cystic fibrosis: a pilot study
Source: Front Nutr. 2024 Sep 25;11:1441201. doi: 10.3389/fnut.2024.1441201 (PMC11462092; doi:10.3389/fnut.2024.1441201)
Supplement: Supplementary file 1 [file Data_Sheet_1.docx]

Supplementary Material

**Supplementary Table 1—DXA Body Composition Data Excluding Participant #2***

|  | **Baseline** | **Post-LGL Diet** | **p-value** |
| --- | --- | --- | --- |
| Weight (kg) | 65.1 ± 3.7 | 64.3 ± 3.5 | 0.23 |
| BMI (kg/m^2^) | 24.1 ± 0.7 | 23.7 ± 0.6 | 0.22 |
| Total fat mass (gm) | 23173 ± 3096 | 21849 ± 3273 | **0.05** |
| Total lean mass (gm) | 44307 ± 4379 | 44392 ± 44493 | 0.86 |
| % fat mass | 33.6 ± 4.2 | 32.3 ± 4.5 | **0.02** |
| Trunk fat (gm) | 10405 ± 1668 | 9746 ± 1733 | **0.05** |
| % trunk fat | 30.4 ± 4.2 | 29.2 ± 4.6 | **0.05** |
| Fat mass index (FMI, kg/m^2^) | 8.5 ± 1.2 | 8.1 ± 1.3 | 0.08 |
| Lean mass index (LMI, kg/m^2^) | 15.8 ± 0.9 | 15.9 ± 1.0 | 0.56 |
| Appendicular lean mass index (ALMI, kg/m^2^) | 6.5 ± 0.5 | 6.6 ± 0.5 |  |

**Participant #2 stopped exercising 4-6 times weekly mid-study*

*Bolded values represent p <0.05*

**Supplementary Table 2—Biochemical Data**

|  | **Pre-LGL Diet** | **Post-LGL Diet** | **p-value** |
| --- | --- | --- | --- |
| **ESR (mm/h)** | 15.9 ± 6.9 | 13.7 ± 5.3 | 0.77 |
| **CRP (mg/L)** | 0.16 ± 0.06 | 0.20 ± 0.07 | 0.26 |
| **HbA1c (%)** | 5.7 ± 0.2 | 5.7 ± 0.2 | 0.55 |

**Supplementary Table 3—Detailed Participant Weight Data**

| **ID** | **Start Weight**  **(kg)** | **End Weight**  **(kg)** | **Lowest Weight**  **(kg)** | **Absolute Change (kg)** | **% Change** |
| --- | --- | --- | --- | --- | --- |
| 1 | 55.2 | 55.6 | 54.9 | 0.4 | -0.7 |
| 2 | 61.4 | 61.8 | 59.7 | 0.4 | 0.6 |
| 4 | 78.0 | 74.1 | 72.8 | **-3.9** | -5.0 |
| 5 | 60.3 | 61.2 | 61.2 | 0.9 | 1.5 |
| 6 | 49.0 | 49.7 | 49.2 | 0.7 | 1.4 |
| 7 | 69.3 | 69.2 | 68.0 | -0.1 | -0.2 |
| 8 | 56.0 | 55.8 | 55.8 | -0.2 | -0.4 |
| 9 | 80.5 | 81.2 | 81.7 | 0.7 | 0.9 |
| 10 | 75.3 | 73.3 | 72.4 | **-2.0** | -2.7 |
| 11 | 62.7 | 58.3 | 58.3 | **-4.4** | -7.0* |
| **Average** | **63.7** | **63.6** | **62.9** | **-0.8** | **-0.5** |

**After study completion, participant 11 disclosed intentionally trying to lose weight mid-study*

**Supplemental Figure 1— Diet Tolerability Questionnaire**

**
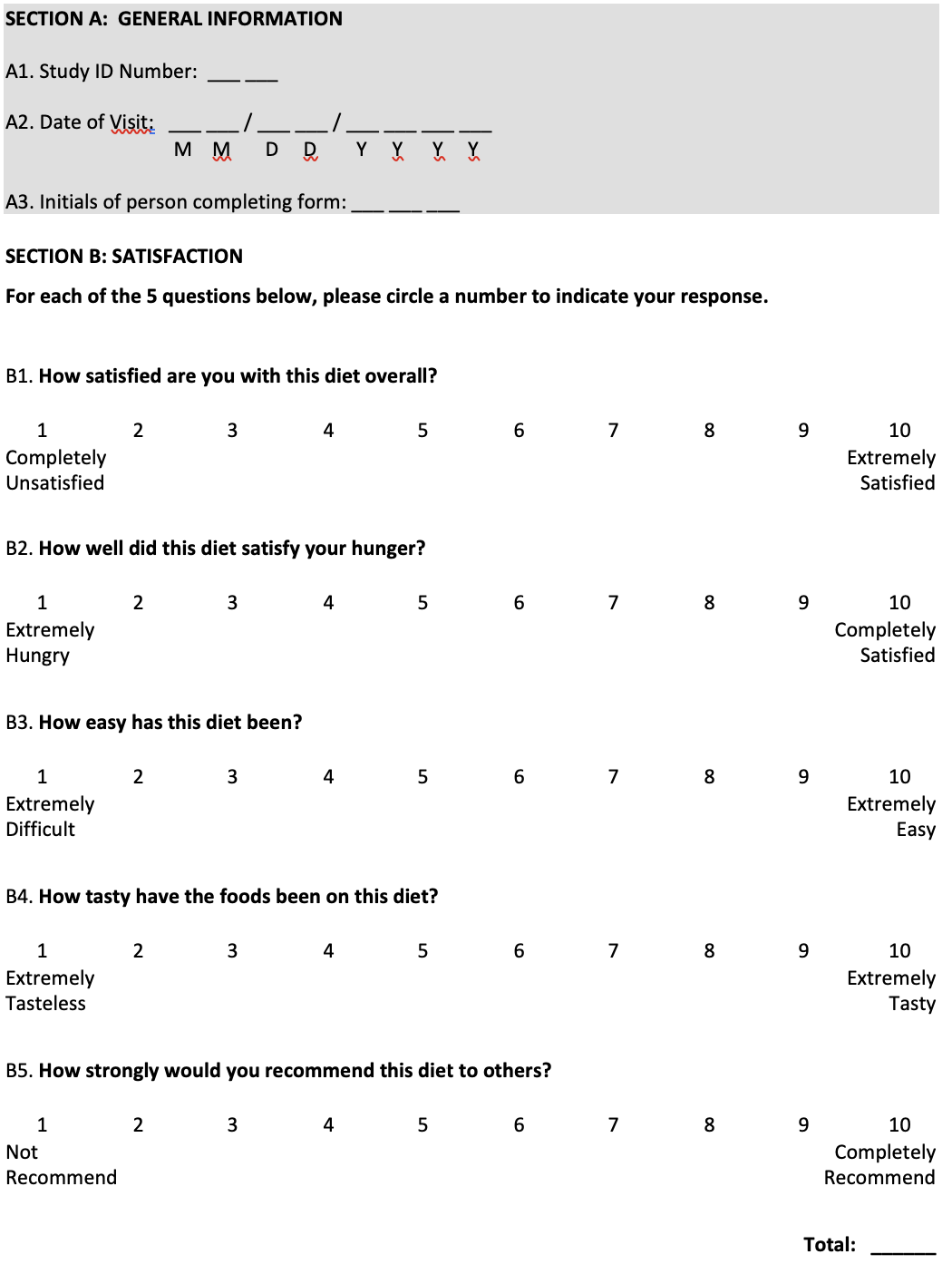
**

**Supplemental Figure 2— Study Timeline Diagram**:


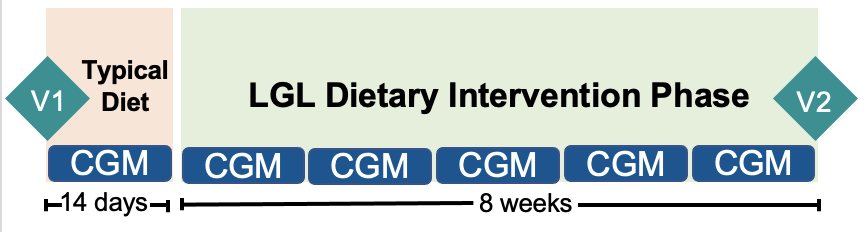


*CGM, continuous glucose monitor; V#, visit number*
